# Developing leaders from within: a thematic analysis of allied health professionals’ experiences in a fellowship programme

**DOI:** 10.1186/s12909-026-09367-5

**Published:** 2026-05-07

**Authors:** Alice Thompson, Robyn Jones, Yitka Graham

**Affiliations:** Helen McArdle Nursing and Care Research Institute, University of Sunderland, Sunderland, England, UK

**Keywords:** Leadership, Development, Transferable skills, Community, Barriers, Experiences

## Abstract

**Background:**

Allied health professionals play an integral role within the NHS because of their input into the delivery of high-quality patient care and help meet the demand of the workforce. However, allied health professionals remain underrepresented in senior leadership positions. The evidence suggests that there is limited access to development opportunities, which impacts the retention and supply of new Allied Health Professionals. Fellowship programmes have been developed as a means of providing learning opportunities for allied health professionals to develop strategic leadership skills and system-level thinking. The current study aims to explore the effectiveness of the NENC Allied Health Professions (AHP) Clinical Fellow Workforce Programme by gaining an understanding of participants’ experiences with the programme and what impact this has had on their professional development, identity and career aspirations.

**Method:**

A qualitative approach involving semi-structured interviews with 9 participants in the fellowship programme was employed. The interviews were audio recorded and transcribed verbatim. The data were analysed thematically by each member of the research team, and investigator triangulation was used to reach a consensus of themes among the research team. The findings were validated by participants to ensure that they accurately reflected their experiences engaging in the programme.

**Results:**

The research team identified themes that reflect the experiences of fellows in the programme, including two overarching themes: “Growth and Challenges in the Fellowship Experience” and “Developing AHPs: Bridging Expertise to Leadership”, each with associated key themes and subthemes. Fellows recognised the opportunity for professional development and emphasised the supportive environment driving their success. Fellows developed leadership and networking capabilities, preparing allied health professionals to develop more leadership positions across the NHS workforce.

**Conclusions:**

The findings provide valuable insight into fellows’ experiences of participating in the NENC AHP Clinical Fellowship Programme, where the findings illustrate the perceived value of fostering leadership capabilities, professional development, and wider system understanding. Further research is needed to establish whether future fellowship programmes as a career development opportunity, translate into sustained increases in AHP representation in senior leadership. In doing so, future research should move beyond self-reported learning to examine how fellowship participation translates into measurable changes in leadership representation, workforce retention, and service delivery.

## Background

Allied health professionals (AHPs) constitute the third largest workforce within the NHS, comprising 185,000 AHPs in fourteen professions who work as autonomous practitioners and are educated to at least the degree level standard [1]. AHPs are seen as a vital group within the NHS and in the NHS long-term plan (2019) and are recognised for their input in enabling the delivery of high-quality patient care and helping the NHS meet demand. Despite their integral role, AHPs remain underrepresented in senior leadership positions, with limited access to structured leadership development pathways [2]. Existing opportunities often prioritise nursing or medical professions, perpetuating a gap and constraining AHP career progression. Therefore, it is acknowledged that there are limited schemes or programs available to prepare more AHP to progress into leadership positions [3].

In response, the Allied Health Professional Strategy for England [4] acknowledges the transformative potential of the AHP community. Part of achieving this potential and addressing the leadership and innovation gap is ensuring an effective supply of new AHPs and the retention of existing staff [5]. Facilitating leadership and career development opportunities at the system-wide level is recommended to address the lack of AHP voice at the senior level and promote retention [6]. This investment will subsequently lead to improvements in patient care, collaboration, and innovation across the workforce; The AHP Strategy for England: AHPs Deliver (2022–2027) acknowledges the transformative potential of the AHP community and positions the strategy as a catalyst for change. Facilitating leadership and career development opportunities at system-wide, board, and executive levels is explicitly recommended to elevate the AHP voice in senior leadership and promote retention [7]. Such investments in leadership have been shown to foster innovation, collaboration, improved care, and better workforce engagement across the AHP community [8].

Fellowship programmes have emerged as a key mechanism for addressing the leadership gap among allied health professionals. Fellowship programmes provide structured, experiential development by seconding AHPs for a fixed period to lead strategic projects, receive mentoring, and gain exposure to system-level decision making. This approach enables participants to apply their clinical expertise in new contexts, building strategic leadership capacity and professional networks [8]. It is suggested that fellowship programmes that are tailored for AHP provide experiential learning opportunities to develop strategic leadership skills and networking at the system level [9]. This is supported by the findings of previous research, suggesting that AHPs who have participated in existing fellowship schemes have reported an increase in professional skills, including increased confidence, enhanced professional networks, strategic thinking and leadership and project management skills [9, 10]. However, despite these benefits, the AHP continues to face challenges in translating leadership learning into its everyday role, particularly where hierarchical structures are in place or where cultures inhibit innovation and development [11].

Despite logistical challenges, Health Education England has emphasised the importance of developing AHP leadership as essential for a future-ready workforce [12]. Fellowship programmes play a key role not only by enhancing leadership skills but also by fostering self-efficacy through supportive, collaborative communities [13]. These initiatives enable AHPs to share experiences, access mentorship, and build networks across organisational boundaries, creating a strong sense of professional belonging. Research shows that such programmes promote visibility, cross-disciplinary learning, and influence within system-wide trusts [14, 15]. The community-building element of fellowship programmes includes working on system-wide projects, with opportunities to collaborate across systems and encourage shared leadership and innovation [16]. These communities of practice help leadership behaviours transition into AHP roles and embed AHP perspectives into strategic decision-making [17, 18].

### AHP clinical fellow workforce programme NENC ICB

Recognising the ongoing barriers to professional development within the AHP workforce, the NHS Long-Term Workforce Plan (2023) outlined workforce improvement priorities for employers to know, grow, recruit, and retain a sustainable AHP workforce for the future. Within the workforce plan, there are three clear priorities for the AHP: train, retain and reform. Without all these priorities, educators have a clear role in enabling sustainable progress and ensuring the development of a workforce so that it can meet the needs of the NHS.

In 2024, the North‒East and North Cumbria Integrated Care Board (NENC ICB) funded several six-month Allied Health Professional (AHP) Clinical Fellowships. The clinical fellowship programme was designed and developed by focusing on the improvement areas outlined in the NHS Long-Term Workforce Plan (2023). The main aim of the clinical fellowship programme was to provide opportunities for AHP to develop leadership skills, alongside the delivery of a project that encompassed the NENC system. Each of the clinical fellows was on a flexible six-month secondment from their AHP role. Clinical fellows were expected to set their own goals within the boundaries of the individual workstream and integrate their project into the wider AHP workforce programme. The clinical fellows undertook specific projects, which were mapped onto KPIs outlined in the NENC AHP Workforce Priorities 2024–25. This enabled AHPs to step beyond their clinical roles and contribute to system-level change to undertake strategic projects within their subsequent NHS trusts and Integrated Care Systems.

### Learning through experience: theoretical perspectives

Understanding how AHPs learn and develop through fellowship programmes benefits from a clear theoretical framework. To guide interpretation, this study draws on Kolb’s Experiential Learning Theory (ELT; [19]), which emphasises learning as an iterative cycle of experience, reflection, conceptualisation, and experimentation. ELT provides a cyclical model in which knowledge is constructed through experience, reflective observation, abstract conceptualisation, and active experimentation. Fellowship programmes align closely with this cycle, offering the opportunity to be reflective of the experience. In line with ELT, the NENC AHP Fellowship programme offered protected time away from clinical duties to engage in leadership activities, encouraging participants to move through the learning cycle by reflecting on these experiences, conceptualising new approaches to leadership, and testing them within strategic projects.

The social dimension of learning is equally important. Wenger’s concept of communities of practice (CoP) provides a useful lens through which to view the collaborative and relational aspects of leadership development in AHP [20]. In the context of the NENC AHP Clinical Fellowship Programme, fellows are embedded within temporary but purposeful communities, consisting of multidisciplinary HDHs, across various NHS trusts and organisations, allowing shared learning to occur. This framework helps to conceptualise how fellows’ identities and practices develop through collaboration in these communities. The CoP also helps to explore how leadership learning is sustained beyond the programme, as fellows utilise the learning community by developing insights and drawing upon them in their AHP roles. By integrating these theoretical perspectives, researchers can interpret the learning processes of participants, helping to conceptualise how participants have developed skills and engaged in professional transformation [21]. This will support a deeper exploration of how leadership capacity is cultivated in the AHP workforce and how fellowship programmes can be optimised for sustainable impact.

### Current study

While there is growing recognition of the value of fellowships in developing AHP leadership, there remains limited research on the effectiveness of fellowship programs, how they function across diverse contexts and systems, and their impact on participants. There is a need for research to understand the experiences and impact of AHP’s learning and leadership development in such programs. The aim of the current study is to address these gaps by exploring the effectiveness of the NENC Allied Health Professions (AHP) Clinical Fellow Workforce Programme, specifically by gaining a deeper understanding of participants’ experiences and reflections on their learning. This study addresses the following research question:

- RQ: *How do AHP Clinical Fellows experience fellowship programmes, and what impact do these experiences have on their professional development, identity, and career intentions?*

By centring the voices of the fellows, the study sought to uncover insights into what aspects of the programme supported or hindered their development, which could inform future iterations of similar fellowship programmes.

## Methods

### Design

The study used a qualitative approach, as this approach allows for a deep understanding of the experiences of participants in the programme [22]. A realist approach underpinned the research design; by applying this approach, participants’ accounts were treated as true reflections of their experiences of engaging in the programme, where the patterns of underlying social and structural mechanisms and the contextual factors that could influence them could be explored in the data [23, 24].

### Ethical considerations

The study was conducted in accordance with the NHS Research Governance Framework for Health and Social Care Research as described by the Health Research Authority (HRA), and the ethical guidelines set out by the University of Sunderland’s Research Ethics Committee. Ethical approval from the University of Sunderland was obtained prior to data collection, which was granted on 20/09/2024. Participant documents were presented to participants during recruitment so that they could make an informed decision surrounding participation. Written informed consent was obtained from each participant before data collection.

### Participants and data collection

A purposive sample strategy was employed to ensure diverse representations of fellows across specialties, geographic locations, and career stages. Invitations were sent via email, detailing the purpose of the study and requesting their voluntary participation. Nine out of the twelve participants who took part in the programme agreed to take part in the research, representing 75% of the total fellowship cohort (see Table 1). Given the relatively small cohort of fellows within the programme, the sample size obtained was deemed sufficient, as participants provided rich and detailed accounts of their experiences. Data saturation was considered to have been reached when no new themes or insights emerged during the later stages of analysis.

**Table 1:** Participant demographics

Participant	Position/Band	Age		Gender	Race/Ethnicity	AHP Role
1	Fellow (Band 7)	40		Female	White	Senior Physiotherapist
2	Fellow (Band 7)	41		Female	White	Advanced Occupational Therapist
3	Fellow (Band 7)	36		Female	White	Occupational Therapist
4	Fellow (Band 7)	47		Female	White	Operational Lead Physiotherapist
5	Fellow (Band 5)	37		Female	White	Occupational Therapist
6	Fellow (Band 7)	44		Female	White	Speech and Language Therapist
7	Fellow (Band 5)	51		Female	White	Physiotherapy Associate Practitioner
8	Senior Fellow (Band 8)	44		Female	Asian/Asian British	Senior AHP Clinical Fellow/AHP Workforce Development
9	NENC AHP Faculty Integration Lead (Band 8)	41		Female	White	AHP Support worker Lead & Occupational Therapy

**Fig. 1:**
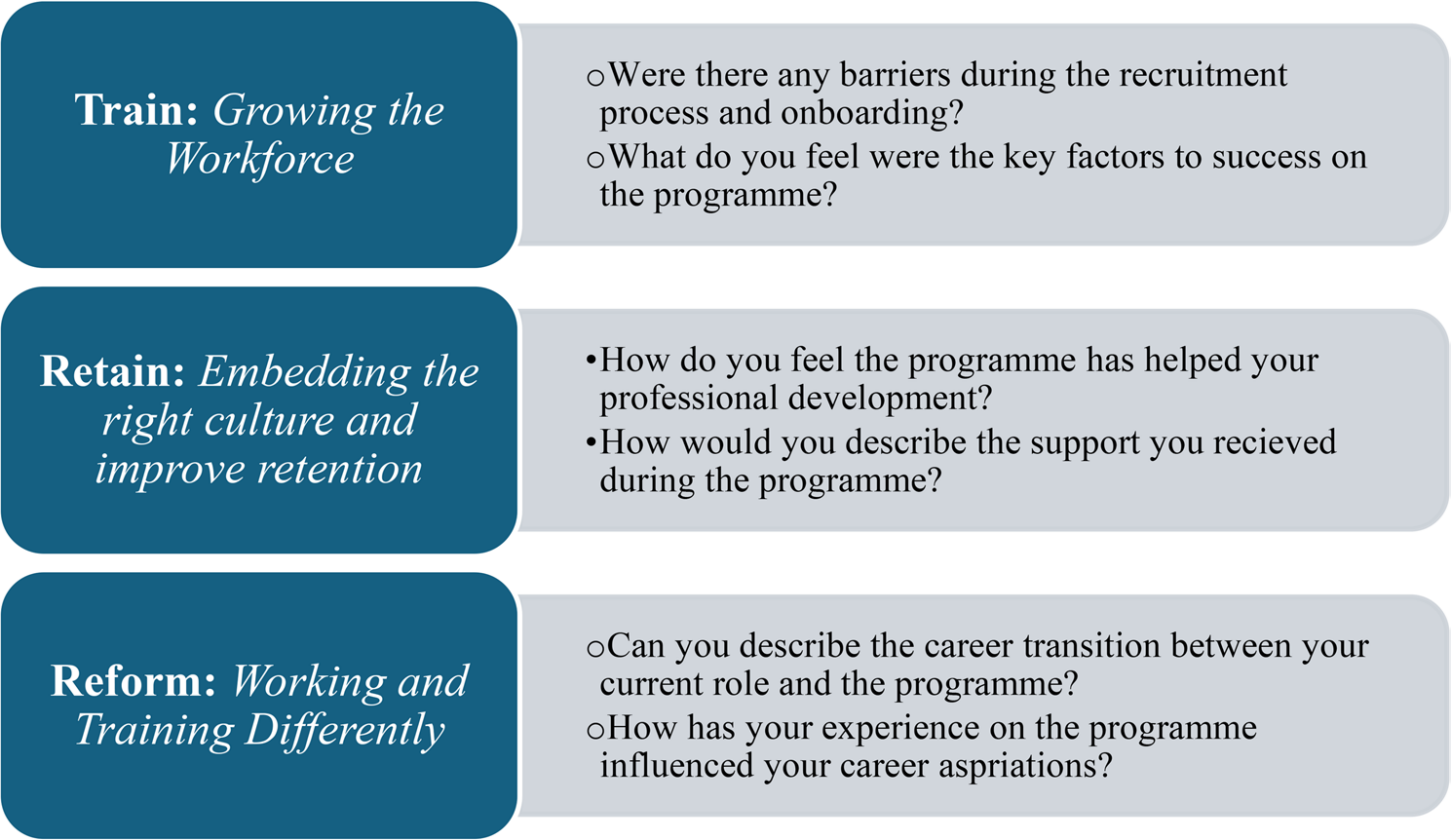
Examples of open-ended questions mapped onto key priority areas

Data collection took place from September 2024 to November 2024. Semi structured individual interviews were the chosen format for data collection. Interviews commenced four weeks after the fellowship programme had concluded. This form of data collection was chosen on the basis of participant preferences and their proven efficiency in facilitating confidential and insightful discussions [25]. The interviews were conducted via secure online video conferences such as Microsoft Teams, each lasting between 45 and 60 min. A topic guide was developed from the NENC AHP Workforce Priorities 2024–25, with open-ended questions designed to cover participants’ experiences of the key priority areas; see Fig. 1 for example questions. The topic guide was used to guide and encourage discussion, ensuring consistency across all interviews while allowing for flexibility in exploring participants’ unique experiences [26]. Questions were also adapted through slight rephrasing to capture the experiences of the different representations of roles in the programme. With participants’ consent, interviews were audio-recorded via encrypted devices to ensure data security. Audio recordings were transcribed verbatim by one member of the research team, including non-verbal elements such as pauses, emphasis, and emotional expressions where relevant to meaning. Transcription followed an iterative process, whereby transcripts were checked against audio recordings for accuracy. All identifying information was removed during transcription to ensure anonymity. The researcher engaged in active familiarisation with the data during transcription, which supported early immersion in participants’ narratives and informed subsequent coding. Audio recordings were deleted following transcription.

### Data analysis and analytical rigour

The data were analysed independently by each member of the research team (AT, RJ, YG) via the six phases of thematic analysis (TA) described by Braun and Clarke [27]. The interview transcripts were read several times, and initial observations were made, where initial codes were generated across the entire dataset (Phases 1 & 2). Codes were generated inductively (data-driven) to understand the experiences of the fellows taken part in the programme [28]. Semantic codes were generated to capture the explicit meanings apparent in the data. Latent coding was also used to understand the underlying ideas and assumptions which shaped the patterns apparent within the data [29]. From this process, a total of 186 codes were identified. All initial codes were subsequently collated into potential themes, which initially consisted of 5 large themes. Potential themes were then reviewed and refined into the final themes described within the results (Phases 3 & 4). Finally, detailed descriptions of each theme were developed and complemented by in vivo quotes extracted from the interview transcripts, producing a final report of the findings of the analysis (Phases 5 & 6).

To enhance the analytical rigour and credibility of findings, the strategies of triangulation and member checking derived from Lincoln and Guba’s (1985) framework for establishing trustworthiness of qualitative inquiry were applied during data analysis and interpretation [30]. Both data source triangulation and investigator triangulation took place. First, data source triangulation took place by collecting data from multiple participants across roles, bands, and demographics to capture a diverse range of experiences and viewpoints. This approach helps deepen understanding, clarify meaning, and verify the consistency of interpretations [30].

During the analysis process, investigator triangulation was employed through peer debriefing and collaborative coding within the research team [30]. Involving multiple researchers in the interpretation of transcripts enhanced the credibility of the findings, as emergent themes were regularly discussed, alternative interpretations were offered and explored, and individual researcher assumptions were challenged [30]. This process continued until a consensus on themes was reached among the research team [31]. During the final stages of the research process, member checking, also referred to as participant validation, took place [30]. The preliminary findings of the research were presented to the participants, who were given the opportunity to provide feedback on the interpretations and the extent to which they accurately reflected their experiences of engaging in the programme.

## Results

Across interviews, participants engaged in active reflection on their personal and professional transformation throughout the fellowship, with many describing a shift in how they understood their role, capabilities, and future trajectory. This reflection often centred around increased self-awareness in both personal and professional capacities and appeared to act as an important mechanism through which participants consolidated their learning, occurring both during the fellowship and retrospectively following its completion.

*“Looking back*,* I don’t think I realised at the time how much I was developing… now I can see how my thinking has changed.”* – Fellow.

The findings of this study are presented in two overarching themes, each addressing a key aspect of the programme: “Growth and challenges in the fellowship experience” and “Programme Impact: Bridging Expertise to Leadership”. These themes capture not only participants’ experiences of the fellowship but also their reflections on how these experiences shaped their learning, professional identity, and future career intentions. Each key theme is discussed in detail; associated main themes and subthemes are also identified, which explore the more specific experiences, perceptions, and reflective insights of participants involved in the AHP Clinical Fellowship programme (see Table 2).

**Table 2:** Thematic analysis included the overarching themes associated with themes and subthemes

Overarching Theme	Theme	Subtheme
1. Growth and challenges in the fellowship experience.	1.1. Nurturing Excellence	1.1.1. Team Collaboration & Support
		1.1.2. Expanding Network
	1.2. Navigating Uncertainty	1.2.1. Ambiguous Goals & Expectations
		1.2.2. Logistical Challenges
		1.2.3. Barriers to Support
	1.3. Differing Perspectives Across Roles	
2. Empowering AHPs: Bridging Expertise to Leadership	2.1. Developing Key Practical Skills	
	2.2. Confident Leadership	
	2.3. Inspiration for Career Growth	
	2.4. Fostering a Vision for Change	

### Overarching Theme 1: Growth and challenges in the fellowship experience

The fellowship experience was consistently portrayed as more than just a structured academic program; it was an immersive journey that contributed significantly to personal and professional growth. Three key themes were developed: nurturing excellence, navigating uncertainty and differing perspectives across roles, with associated subthemes.

#### Theme 1.1: Nurturing excellence

**Fig. 2:**
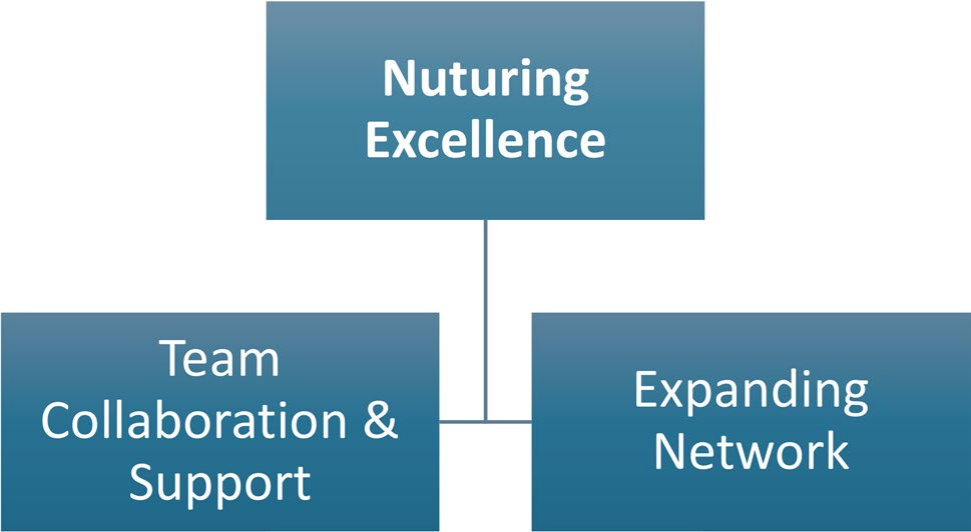
Thematic map of theme 1.1, which explores participants’ experience with the fellowship program

The fellowship not only provided opportunities for skill development, but it also created and environment which fostered a culture of continuous improvement, accountability, and support (see Fig. 2).

Participants described the support and guidance provided to not only have allowed them to achieve a high standard in their individual work, but also for the cultivation of a collective ethos of improvement and mutual support. Importance was placed on both personal and professional growth, where fellows described this to have allowed for them to nurture one another, highlighting the collaborative and supportive community developed during the programme. Participants described excellent pastoral support provided by leadership and senior fellows, which was used as a primary example of such nurture, positively impacting development in the programme.

*“I felt that she was such a supportive figure. She was always very accessible*,* and when I sent her any questions via email*,* she’d get back truly quickly*,* which was brilliant.”* – Fellow.

*“I think J and M were truly approachable. I always felt very positive after speaking to them and they kind of acknowledged that the work I had been doing it was probably different to everybody else’s and it was part of that process*,* you know*,* of networking and trying to understand how things work and what I needed to do.”* – Fellow.

Participants consistently highlighted the impact of having access to research support from the University of Sunderland. This support was not only perceived as a valuable resource, but participants also frequently described this as an indispensable asset throughout the research process, providing critical support for the successful completion of projects.

*“I think the research talks have been truly helpful*,* particularly around writing up the project and just thinking about the data truly.” – Fellow [speaking about research support]*

"I think even having access to the university is massive. You know, we’ve got contacts such as research support and stuff within the hospital, but they’re not as readily available as the University of Sunderland has been."

Within this theme, two subthemes were developed, accounting for more specific experiences expressed by participants:

##### Subtheme 1.1.1. Team collaboration & support

Participants placed a strong emphasis on team collaboration. Collaboration appears to be not only an avenue for shared learning among fellows, but it could also act as a driver for building strong, supportive relationships and a sense of community. Many participants described working within a team, which had a significant effect on the success of their project, through the exchange of ideas and problem solving as a collective effort.

*“I think probably between the four of us*,* we probably managed to achieve more from our four brains working together than we would have all been able to achieve individually working in those projects*,* so that has been truly nice.” – Fellow*.

*“I think a sense of community for me has definitely come from the smaller group.” – Fellow*.

##### Subtheme 1.1.2. Expanding network

The fellowship programme provided numerous opportunities for participants to connect with a wide range of individuals, including the wider system and the AHP community. For many, this experience was one of the most valuable aspects of the program. Participants emphasised the importance of how building external connections allowed them to access innovative ideas, resources, and opportunities, fostering continuous growth and excellence. The ability to expand their network appeared to not only have professional benefits, but also personal, where they described an enhanced sense of belonging and self-confidence.

*“Networking. I mean*,* I Iove networking anyway*,* but again I’ve been able to speak to some amazing people because you’ve been linked in with established and credible individuals*,* this if I was just a clinician*,* somebody wouldn’t necessarily have picked up this random email off me saying “oh can I have a chat.” They might have done.”* – Fellow.

#### Theme 1.2. Navigating uncertainty

**Fig. 3:**
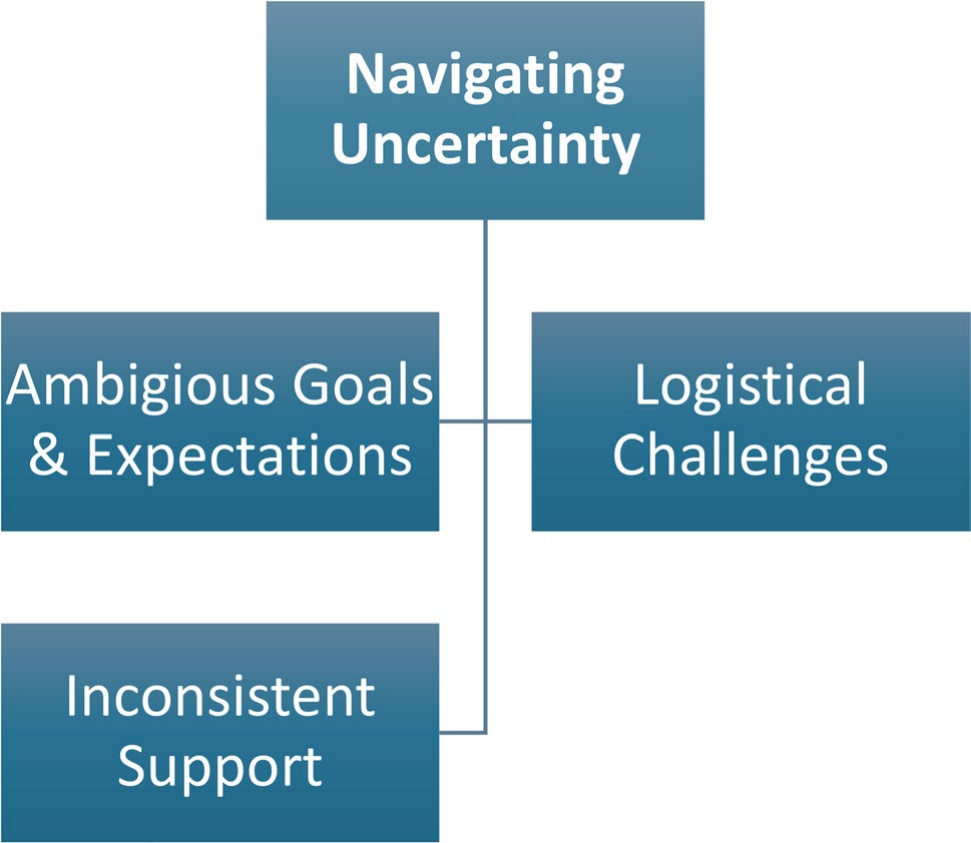
Thematic map of theme 1.2, which explores participants’ experience with the fellowship program

Experiences of uncertainty were frequently described by fellows, caused by a lack of clear direction, structure, or guidance to certain aspects of the fellowship programme. Such experiences of uncertainty led to feelings of overwhelm and confusion, negatively impacting their fellowship experience. Within this theme, three additional subthemes were identified (see Fig. 3).

*“I definitely felt overwhelmed… At that point*,* I felt truly overwhelmed*,* and I didn’t truly know what to do because there wasn’t any structure. Therefore*,* they were just like*,* OK*,* go and do these projects.”* – Fellow.

##### Subtheme 1.2.1: Ambiguous goals & expectations

A common experience described was the struggles surrounding the unclear aims and expectations of the programme, where a lack of clear direction during the initial stages caused significant frustration for participants. The program’s unclear structure and ambiguous expected aims and outcomes created uncertainty in both the projects and professional development. The participants expressed confusion about how to effectively navigate their work, which caused anxiety.

*“However*,* my parameters were so wide at the beginning*,* I did feel like… I do not know what the word is. Like a little fish in a big pond and was kind of like*,* “Where the hell do I start here?” and that fear of doing the wrong thing… I did feel like I was very different to everybody else in the fact that everyone else had truly clear parameters and I did not*,* and that made me feel like… at the beginning*,* I constantly felt like I was behind everybody.”* – Fellow.

For individuals working alone, the absence of a defined path proved particularly challenging, where they frequently expressed feeling unsupported and directionless. However, such feelings were mitigated for those working within a group, where the collaborative environment allowed for peer discussion and clarification.

*“Oh*,* it’s so helpful to have our group working*,* but I don’t think a lot of other people have had that. I think that must have been a lot more challenging for people.”* – Fellow.

Despite these initial frustrations, several participants acknowledged that the open-ended nature of the programme allowed for freedom and personal development. Upon mainly retrospective reflection, they expressed understanding that the absence of preestablished goals was intentional, which intended to encourage them to take ownership of their projects, allowing for the application of their individual ability and creativity to achieve meaningful outcomes.

*“I was in a lucky position with quite a structure programme of things to do*,* so I was able to kind of just work through that… I still sort of set my own goals.”* – Fellow.

Therefore, although such experiences initially caused uncertainty and discomfort for many, participants frequently felt that it was this freedom and flexibility that made the fellowship so effective in preparing them for future success in managing projects and impactful decision-making. The open-ended nature of the programme provided them with a valuable learning experience that pushed them to step outside of their comfort zones, challenge their assumptions, and cultivate skills they could carry forward into their careers.

##### Subtheme 1.2.2: Logistical challenges

Significant barriers related to line management support and the time constraints of the fellowship programme were identified, which participants frequently identified to hinder their progress. A key challenge arose from the lack of support from line management in their clinical/full-time role, where several fellows felt as although their line managers did not fully endorse their involvement in the programme. When line managers did not see the potential value of the fellowship and how this could enhance the fellow’s role, it became more difficult for participants to receive the necessary practical support and emotional encouragement, as echoed in the quotes below.

*“My line manager at the time*,* wasn’t keen for me to do the fellowship because she couldn’t see what it would bring back to the team…I think you’re starting from a base where my line manager wasn’t truly engaged in it*,* which probably didn’t help”* – Fellow.

*“So*,* I think that my direct line manager has not been fully on board with this project*,* she hasn’t been particularly interested in it… she said… you need to stay*,* and you should be building up your clinical skills.”* – Fellow.

The structural timing of the programme was also presented as another logistical challenge. Fellows dedicated only one day per week to the fellowship over a span of six months. While acknowledging that the fellowship’s purpose is to build ability, participants reflected on the difficulty of fitting a comprehensive and impactful project into a constrained time limit without prior experience or a fully developed brief. Participants expressed limited time to have created a high-pressure environment, which led to finding opportunities for meaningful collaboration and key stakeholder engagement to be challenging, especially for individuals who expressed already managing demanding roles.

*“However*,* also*,* because I’ve truly tried to embrace that collaborative side to it and when you are one day a week and trying to include frontline clinicians and strategic*,* whoever they are*,* system-level people*,* and trust-level people who are very busy*,* trying to coordinate that on one day a week to make sure you have their input and you’re listening to what they’re saying and to be able to react to that appropriately. You know*,* I think you’re cutting it fine doing that on six months*,* especially when you have never done this*,* when you did not have those networks already there and you did not necessarily know what you were doing. For the first couple of months… I do not feel like I wasted it but say if I had a tighter brief or had previous experience*,* then I probably would have hit the ground running. However*,* that is the whole point of the fellowship*,* is not it?” -* Fellow.

##### Subtheme 1.2.3: Barriers to support

Although most participants experienced incredibly positive and helpful support, various participants discussed the varying levels of support received, which were acknowledged to have been amplified by certain barriers. Participants suggested that support was affected by delays in senior staff starting their role. Although recognised to be out of the senior staff’s control, without clear structures or readily available mentors, participants experienced feeling frustrated and isolated, leading to difficulties in navigating the resources that were available to them.

*“The support was there*,* and I don’t it’s hard to comment isn’t it*,* not having understood what it would be like with her in that role. I didn’t feel the support was lacking. It might have felt different if she had been there*,* and the structure might have looked different.”* – Fellow.

The participants also discussed the impact of the remote/hybrid style of work on the support received. The transition to online frameworks often presents barriers to building meaningful relationships, accessing real-time support, and fostering a sense of community. Fellows found virtual engagement to be somewhat isolating, particularly when trying to connect with key stakeholders or mentors. The absence of face‒to-face interactions, which allow for more immediate feedback and stronger connections, exacerbated feelings of isolation for some participants.

*“We tried to combine but they didn’t truly work out*,* so yeah for the last three months it’s felt*,* from a fellowship perspective more isolating for me.” – Fellow*.

Nonetheless, participants expressed that such experiences presented them with opportunities to develop resilience, adaptability, and creativity, where they were able to navigate the programme independently and through community fellows. It could be considered that retrospective reflection allowed for fellows to constructively consolidate their learning and experience of the programme.

#### Theme 1.3: Differing perspectives across roles

Differences in the experiences of individuals in the fellowship program were observed, shaped by their respective banding, role, and responsibilities, revealing insightful distinctions in expectations, day-to-day tasks, and overall contributions to the program. The main differences were observed between Band 5 fellows and Senior Fellows/Management-level.

Band 5 fellows on the programme alluded to feeling overwhelmed in their project teams. There was a sense that the expectations placed upon them were often comparable to those for more senior fellows, such as Band 7 Fellows. This discrepancy between their level of experience and the expected scope of their responsibilities led to feelings of uncertainty and anxiety, also causing frustrations and comparative attitudes among fellows.

*“Sometimes it felt at the beginning*,* I do not know if it was our perception and it wasn’t- maybe how we perceived that*,* it was that*,* yeah*,* we were in the same and we shouldn’t be because we do not have that experience or that knowledge. We are in a totally different band*,* and I do not know*,* I do not know. It felt a little bit at first*,* but as I said*,* I do not know*,* I think things have become easier*,* as well as we were doing the project. Maybe at first it felt a little bit too - big*,* and as you start to do it*,* it feels that is more approachable*,* it’s easier. We can do it. We have the support yeah. However*,* the first impression*,* because we did talk about that*,* it felt like the expectations from us were the same for everyone else.”* – Band 5 Fellow.

Band 5 Fellows discussed how they entered the fellowship program with the understanding that their role would allow them to gradually build experience and responsibility. However, they quickly noticed that the expectations set by the organisation often mirrored those placed on more experienced fellows. The lack of direction surrounding project management led to a sense of pressure to meet expectations that felt disproportionate to their level of experience or training.

*“There was no real direction as to what that was to look like or anything like that. Which I suppose in a sense was up to us to decide and work out*,* but I guess for myself*,* it felt a little bit like the expectation was as high for us in a band five as it was for the band seven clinical fellows.”* - Band 5 Fellow.

The challenge of navigating these elevated expectations was compounded by a sense of reluctance or fear around seeking help from colleagues or mentors, where they reported feeling hesitant to ask for assistance or admitting when they did not know something. Such hesitancy appeared to be caused by feelings of confusion, fear of being deemed as ”uncapable”, and avoidance of increasing others’ workloads.

*“…There wasn’t any differentiation*,* in a sense. Therefore*,* I think maybe… maybe it was partly me as well*,* I didn’t reach out as much for support*,* but I also didn’t truly know what I was asking for.”* – Band 5 Fellow.

Band 5 Fellows’ experiences could reflect a complex and often challenging dynamic within the fellowship program. However, many fellows found ways to cope through initiative-taking communication, seeking peer support, and gradually building mentoring relationships. These efforts helped them find the guidance and reassurance they needed to navigate the complexities of the fellowship and succeed in their roles.

For senior fellows and management, one of the most notable challenges they faced was the balancing act between providing pastoral support and engaging in the project-specific responsibilities that came with their roles. While they entered the fellowship with the expectation that they would have a key role in supporting the development and well-being of their peers, their day-to-day responsibilities often evolved in unexpected ways, pulling them into more tactical and operational duties. Alongside conflicting responsibilities, this was also accentuated by management during the startup process of designing the programme.

*“What my understanding is to provide pastoral support. Therefore*,* not truly about what they were delivering in their projects*,* but just to check from a well-being point of view that they are feeling supported*,* that they are developing and getting something out of the fellowship. Whereas the actual project deliverables*,* that was more [manager] initially. I think the real challenge around that is it’s hard to separate the two.”* – Senior Fellow.

*“I was coming up with people who were also trying to find their feet with new processes*,* and me*,* without knowing what a normal process would be like anyway.”* – Programme Lead.

### Overarching Theme 2: " Developing AHPs: bridging expertise to leadership”

This overarching theme captures the profound influence the programme has had on participants, professional development, and the role of the AHP. The impact of the programme was discussed among participants in relation to their personal and professional development, emphasising how their skill development in the programme directly affects their ability to provide impactful work.

**Fig. 4:**
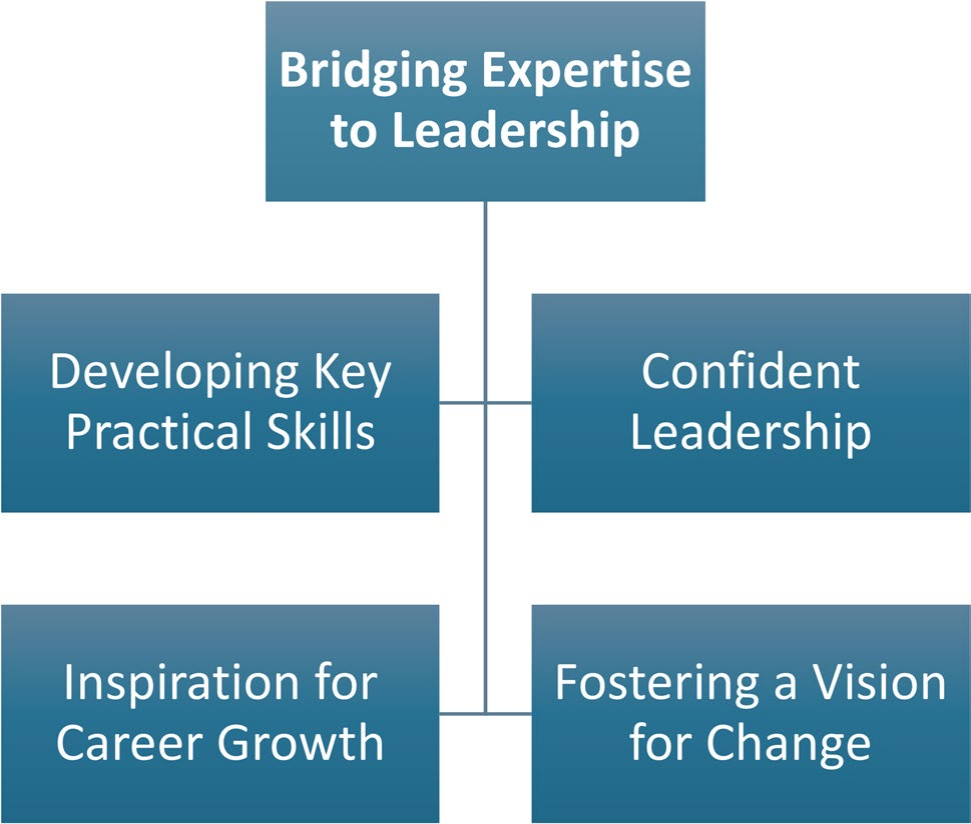
Thematic map of participant skill development across the AHP fellowship program

“Bridging expertise to leadership” refers to the wide variety of skills and knowledge that the programme has impacted, leading to the development of a range of practical and transferable skills, as well as the ability to aid in self-growth and development. The themes identified within this overarching theme reflect participant’s perceptions of improved skills, knowledge and technical and transferable skills developed through the fellowship, which they perceive to enhance clinical practice. The programme appeared to equip participants with the tools, networks, and confidence needed to thrive in their professional roles while also providing them with the experiences and mindset necessary for them to step into leadership positions. See Fig. 4 below for overarching theme and associated themes.

*“Therefore*,* I think*,* actually*,* it’s challenging our perceptions*,* a little bit*,* to think about how we can do things differently.”* – Fellow.

#### Theme 2.1: Developing key practical skills

Fellows discussed how they have developed key transferable practical skills, from analytical skills to the ability to communicate effectively, which could be applied to their current role within the NHS, enhancing their performance.

*“You know*,* I’ve developed*,* like*,* a more kind of data way of thinking. Looking at the numbers and translating those*,* rather than just*,* like*,* the background behind why we’re doing what we’re doing. Therefore*,* yeah*,* it’s helping me look at things more broadly and deepen my knowledge*,* which then helps me to give a more informed response when I’m communicating with people.” -* Fellow.

#### Theme 2.2: Confident leadership

The participants discussed how their confidence developed significantly across the programme, enhancing their leadership skills and ability to inspire and influence others in a clinical setting. Despite the programme having had put participants into overwhelming situations as described earlier, such situations encouraged fellows to step out of their comfort zone, where they implemented the leadership skills they had developed, increasing their confidence.

*“Therefore*,* I think definitely I feel more kind of confidence in terms of knowledge of how things are working and what’s happening and to set something up and deliver on it and facilitate you know*,* different discussions.”* – Fellow.

#### Theme 2.3: Inspiration for career growth

The participants highlighted how the programme provided tangible steps for advancing their careers, from improving their leadership skills to expanding their professional networks. This initiative-taking approach to career development, often underpinned by ongoing support from the programme, helped participants develop a clearer sense of direction for their future. By combining inspiration for career growth with a commitment to change within their current role, participants described the programme to have empowered them to see themselves not just as experts within their project, but also as future leaders in the AHP network and system wide.

*“I would be so interested in a similar opportunity to this. I’d really like to do more of a senior clinical leaders’ course… just to get that kind of proper leadership course. I think it is the thing that yeah*,* I’ would really like to develop next. I’ve been looking into opportunities for those. I think I’d be keen to take the next step in terms of just leadership.”* – Fellow.

*“It’s kind of given me the drive to develop further in*,* around the leadership skills for supporting the wider support workforce that we’ve got within the organisation. In addition*,* helping them gain those skills to grow in their careers.”* - Fellow.

Despite a strong drive for leadership positions within the fellows, this aspiration was not solely leadership driven. The participants acknowledged the advantages they gained from engaging in the programme and how it allowed them to develop themselves generally; however, from their experience, they were driven to continue this professional development.

*“I think I want to do something that makes a difference to people. That’s truly the main aim*,* isn’t it? We want to be able to develop ourselves. It doesn’t have to be*,* ‘I want to be in leadership…’ Everybody wants to do things in different ways and people want different things for their careers*,* I would say I’ve got what I wanted from it.”* - Fellow.

#### Theme 2.4: Fostering a vision for change

The programme helped refine participants’ vision for change, offering tools and frameworks to apply their learning and ability in ways that have the potential to solve larger system-wide problems. The participants discussed how they were encouraged to expand their scope of influence, as the programme helped them think beyond the confines of their immediate work environment. This system-level thinking appears to be instilled in participants moving forward, encouraging them to identify opportunities for systemic change through leadership.

*“I think that’s one of the key things as fellows*,* that we’ve had opportunity to bridge that gap between something that could have just sat up here*,* as a thing for people to read and do; we’ve kind of applied it and made it much more system-wide and applicable to the work that we’re doing.”* – Fellow.

*“Equally*,* like I said*,* having that systems knowledge or knowledge of that bigger picture helps me do my role well. Therefore*,* I think there’s definitely synergies between the two roles that worked truly well.”* – Senior Fellow.

## Discussion

This exploratory qualitative study examined the experiences of allied health professionals (AHPs) participating in the NENC AHP Clinical Fellowship Programme. The findings provide a nuanced understanding of how allied health professionals (AHPs) can learn and grow through engagement in a structured, yet adaptive fellowship experience. Through thematic analysis, the research team identified key themes that reflect the experiences of fellows in the programme, providing insights into the processes through which leadership capabilities, professional skills, and systemic understanding may have been developed. To deepen interpretation, the research team drew upon Kolb’s experiential learning theory (1984) and Wenger’s communities of practice framework (1998) [19, 20].

### Growth and challenges in the fellowship experience

Across all the interviews, the participants reported strong satisfaction with the AHP fellowship program, particularly appreciation of the opportunity for professional development and the supportive environment it provided. One of the most significant contributors to participants’ experience was the sense of belonging, which was facilitated through peer support and working in teams. This provided fellows with the opportunity to reflect through the sense of community among programme participants, creating a shared learning space and community of practice [20]. The sense of community among programme participants encouraged a collaborative approach to their success, providing both emotional and professional support.

The participants emphasised the strong support network of the fellowship programme, which helped cultivate an increased sense of innovation and collaboration. This collaboration enhanced the social learning system of the programme by providing an opportunity for participants to network system wide, drawing on wider perspectives by creating new connections in ongoing professional settings [20]. Satisfaction is derived from meaningful work experiences, increased professional recognition, and a sense of contributing to system-level work within the NHS, which fosters deeper commitment. The participants valued the sense of community, aligning with the concrete experience and reflective observation phases [19]; as a result, AHP workers are more likely to stay in the NHS if these opportunities become available more often to conduct system-level work [6].

However, logistical challenges and unclear programme goals disrupted the transition to abstract conceptualisation and active experimentation [20]. Without clear structure or feedback, participants struggled to make sense of their experiences or apply learning effectively. This reflects concerns of Billett (2011), whereby workplace learning is most effective when learners are provided with structured guidance and opportunities to reflect [32]. This lack of support led participants to reflect on needs for clearer guidance, influencing their future conceptual understanding of programme design. However, despite the hesitancy around the lack of structure in the programme, the participants adapted to the agile way of thinking by using their initiative and decision-making skills effectively; such skill development is further discussed in the following theme.

Therefore, although the lack of structure led participants to enhance their initiative and decision-making skills, to avoid distress and ensure the effectiveness of future fellowship programs, programs must be well designed with clear deliverables, implementing continuous feedback to support each stage of the learning cycle [33]. As reflected in the findings, the experiences and perceptions of the programme varied across roles. Differing perceived barriers that participants perceived to hinder their progress in the programme were expressed among participants depending on their band and role. For Band 5 Fellows, the main barriers consisted of the expectations of the programme to be excessive, going beyond the responsibilities that should be placed on them according to their band and experience, causing feelings of overwhelm and anxiety. However, the participants reported overcoming such barriers through communication, peer support, and the cultivation of a shared sense of community. This could once again emphasise the importance of collaborative relationships and shared practices in learning, important pillars of Wenger’s (1998) CoP framework [20].

For senior fellows, barriers mainly consisted of feelings as although their understanding of what their role within the programme would entail having not held true, where they felt forced to juggle between providing pastoral support, engaging in project-specific responsibilities, and dealing with day-to-day unexpected responsibilities that they had not anticipated.

The mismatch in expectations was a prominent experience across participants, although the nature of these mismatches varied according to role-specific responsibilities and assumptions. Comparable experiences have been identified in other qualitative research evaluating similar programmes, which could indicate the need for such issues to be addressed [34]. The programme could benefit from clear and transparent expectations to be defined and provided to all fellows prior to initiating to avoid any confusion or frustrations at later stages. Furthermore, the expectations placed on fellows and the structure of the programme itself need to be realistic and align with their experience and capacity, taking into account the specific responsibilities associated with their role/band.

### Empowering AHPs: bridging expertise to leadership

The varied experiences of participants reflect the program’s success in delivering structured, experiential learning development opportunities that drive leadership and clinical capabilities, aligning with the strategic priorities of the NHS Long-Term Workforce Plan [35]. By providing structured learning, mentorship, and system-wide experience, the programme has empowered AHPs to apply learning directly, supporting both individual development of professional skills and broader system transformation. This aligns with Harding et al. (2024), who argue that fellowships tailored to AHPs provide critical opportunities for strategic leadership development and system-level networking [8]. Providing a participant with hands-on experience in system-wide projects and making strategic decisions is effective in preparing AHPs for leading evolving roles in the NHS workforce [36].

The programme has built workforce capacity by providing fellows with the necessary skills and mentorship to step into leadership roles; for example, participants reported gaining confidence in clinical decision-making, with a stronger understanding of how to integrate leadership into practice and system wide. Involvement in workforce-priority projects provides participants with tangible opportunities to apply learning, strengthening their professional identity [37]. Engagement in impactful projects allowed participants to see direct contributions to systemic improvements, further supporting a more optimistic view of the NHS. This further contributed to increasing levels of confidence, particularly in taking on senior leadership positions, supporting findings from Bryar et al. [9]. In contrast to Eddison et al. (2023), participants began to internalise a leadership identity, emphasising how leadership capacity is cultivated through a structured leadership development pathway [2]. By supporting AHPs in their pursuit of advanced roles and leadership positions, this allows them to develop into more leadership positions across the NHS workforce [3].

Engaging in leadership positions through the fellowship programme has equipped participants with the tools to take on higher-level responsibilities. This has allowed the AHP to reimagine future roles and consider new career pathways [20], allowing the AHP to apply the skills developed in the programme and contribute to workforce demands. This was demonstrated through participants’ ability to translate learning on the programme into system-level improvements and AHP roles [19]. This is supported by Gilbert et al. (2019), who demonstrated the impact of fellowship experience in developing strategic thinking and systems leadership [10]. The opportunity to improve workforce priorities within the NHS in specific projects was a key motivator for participants, reinforcing a voice for AHPs in the workforce [6].

Furthermore, it is important to mention how such programmes benefit not only participants although the development of skills and strategic wider system thinking but also the NHS as a workforce. The participants acknowledged how the programme has allowed them to gain system-level knowledge and leadership skills and become adaptive and resilient to an evolving workstream, which has fostered an encouraging mindset of continuous learning and improvement [36, 14]. Such mindsets and the upskilling of individuals could support the NHS by strengthening leadership capacity and innovation and promoting a culture of improvements across organisations and care systems. The fellowship programme then prepares fellows to contribute to the NHS long-term plan by ensuring that they can remain responsive to future changes and innovations [35, 38].

### Implications and future directions

The findings provide valuable insight into how fellowship programmes support career development opportunities for AHP, contributing to increased representation of AHP in senior leadership positions [2]. However, a limitation of this study was the lack of awareness of the programme through recruitment, which was reflected in the participant sample, all of whom were female and predominantly from clinical roles such as physiotherapy and occupational therapy. Future fellowship programs will need to engage with AHP communities to ensure comprehensive representation of AHPs. As the programme has been instrumental in providing leadership self-efficacy among AHP, it is essential to diversify leadership within healthcare settings [9]. Integrating EDI principles into recruitment and engagement effects will support a more inclusive environment, ensuring that leadership within the AHP reflects the diversity of communities with which they work, providing a voice for the AHP across the NHS workforce [6].

As previously discussed, the findings highlighted several barriers, including logistical challenges and a mismatch of expectations. These findings suggest that the design and delivery of the programme should be optimised to enhance learning and improve the overall fellowship experience. To address these issues, it is crucial that the program’s expectations and structure are clearly defined and communicated upfront to minimise uncertainty and frustration. Additionally, it is important to consider the differing perspectives across roles, ensuring that the programme’s structure remains flexible enough to be tailored to the varying capacities, responsibilities, and needs of the fellows. One potential framework to draw upon is the Allied Health Professionals Preceptorship and Foundation Support Programme [39]. Although this programme is aimed primarily at supporting AHPs during their transition to employment and early career stages, its standardised approach to tailored support could be adapted to enhance the current fellowship programme, maximising both the learning opportunities and the overall experience for fellows.

While this study provides rich qualitative insights into participants’ experiences, the findings primarily reflect self-reported perceptions of learning and leadership development. This limitation should be acknowledged when interpreting the results, as the reported changes in capability and impact represent participants’ subjective accounts rather than independently validated outcomes. Future evaluations would benefit from incorporating mixed methods approaches that combine self-report data with objective measures to build a more comprehensive understanding of programme impact. For example, using the NHS Healthcare Leadership Model Self-Assessment Tool (NHS Leadership Academy, 2013) to measure both perceived change and observed change in participant leadership behaviours. NHS Leadership Academy (2025). Self-assessment tool – Leadership Academy [online] https://www.leadershipacademy.nhs.uk. Available at: https://www.leadershipacademy.nhs.uk/healthcare-leadership-model/self-assessment-tool/.

## Conclusion

This study offers insight into fellows’ experiences of participating in the NENC AHP Clinical Fellowship Programme, where the findings illustrate the perceived value of fostering leadership capabilities, professional development, and wider system understanding. The fellowship created an environment that supported experiential learning and was underpinned by principles of a community of practice, offering opportunities that may enable participants to develop skills relevant for future leadership roles. Despite the barriers experienced by participants, including unclear expectations and a lack of structure, these barriers also presented valuable opportunities for participants to use their initiative, communication, and decision-making skills. Fellows demonstrated their ability to adapt to and overcome such challenges, allowing them to successfully complete the programme while contributing to their personal and professional growth.

However, the findings also recognise that despite such challenges serving as important learning opportunities, improvements to optimise the programme and address the logistical issues identified are necessary to reduce frustrations and anxieties and improve participants’ experiences. Importantly, the upskilling, personal, and professional development that the programme cultivates could benefit the broader workforce of the NHS by enabling individuals to feel more equipped to adapt the continuous learning, innovative, improvement, and change-responsive culture that the NHS long-term plan aims to promote. Building on Harding et al.’s (2024) evaluation of the AHP Leadership Fellowship, our findings reinforce the need to address these gaps. By tackling issues of diversity, access, and consistent design, the full potential of AHP fellowships could be realised, ensuring sustained benefits for individual career trajectories and wider system outcomes. Future research should therefore move beyond self-reported learning to examine how fellowship participation translates into measurable changes in leadership representation, workforce retention, and service delivery.

## Data Availability

All data supporting the findings of this study are available within the paper and its Supplementary Information. All data supporting the findings of this study are available within the paper and its Supplementary Information.

## Abbreviations

AHP’s: Allied health professionals
NHS: National Health Service

## References

[1] CapitalAHP |, NHS England. NHS England | Workforce, training, and education. ; 2023 Mar 1 [cited 2025 May 1]. Available from: https://www.hee.nhs.uk/our-work/capitalahp

[2] Eddison N, Healy A, Darke N, Jones M, Leask M, Roberts GL et al. Exploration of the representation of the allied health professions in senior leadership positions in the UK National Health Service. BMJ Leader. 2023; leader-2023. 10.1136/leader-2023-000737 PMC12038103 37620124

[3] Gibbs V, Griffiths M. AHP leadership in academia: opportunities, challenges, and current positioning. Stud High Educ. 2021;46(11):2216–29.

[4] NHS England. The Allied Health Professions strategy for England: AHPs Deliver. [cited 2025 May 1]. Available from: https://www.england.nhs.uk/ahp/allied-health-professions-strategy-for-england/

[5] Turner A, et al. Exploring the impact of leadership development programmes for Allied Health Professionals. Int J Health Serv. 2020;50(1):68–85.

[6] Mizzi L, Marshall P. Inequitable barriers and opportunities for leadership and professional development, identified by early-career to mid-career allied health professionals. BMJ Lead. 2024; leader-2023. 10.1136/leader-2023-000880 38191238

[7] Eddison N, Healy A, Darke N, Jones M, Leask M, Roberts GL, Chockalingam N. Exploration of the representation of the allied health professions in senior leadership positions in the UK National Health Service. BMJ Lead. 2024;8(2):119–26. 10.1136/leader-2023-000737. 10.1136/leader-2023-000737 PMC12038103 37620124

[8] Harding D, Lycett H, Avery L, Kumaresan T, Madden V. Building allied health professions’ leadership self-efficacy through authentic experiential learning: a participatory evaluation of allied health professions leadership fellow secondments. BMJ Lead. 2024. 10.1136/leader-2024-001079 39349044

[9] Bryar R, et al. Developing leadership capacity in the Allied Health Professions: A narrative review. J Health Leadersh. 2021;14:77–89.

[10] Gilbert A, et al. Fellowship programmes as a means of professional development for AHPs. Br J Healthc Manag. 2019;25(2):90–7.

[11] Nancarrow SA, et al. Ten principles of good interdisciplinary teamwork. Hum Resour Health. 2013;11(1):19. 23663329 10.1186/1478-4491-11-19 PMC3662612

[12] Health Education England. Supporting the development of the AHP workforce and AHP careers. NHS England; [cited 2025 May 1]. Available from: https://www.hee.nhs.uk/our-work/allied-health-professions/supporting-development-ahp-workforce-ahp-careers

[13] Monkhouse A, Sadler L, Boyd A, Kitsell F. The improving global health fellowship: a qualitative analysis of innovative leadership development for NHS healthcare professionals. Glob Health. 2018;14:1–14. 10.1186/s12992-018-0384-3 PMC6050734 30016970

[14] West M, Armit K, Loewenthal L, Eckert R, West T, Lee A. Leadership and Leadership Development in Health Care: The Evidence Base. London: Faculty of Medical Leadership and Management; 2015.

[15] Willis J. Fellowship Schemes and Career Progression: Exploring System Impact for Allied Health Leaders. AHP J. 2021;9(3):43–55.

[16] Health Education England. AHP Leadership Programme Evaluation Report. HEE; 2019.

[17] Scanlon D, Atkinson T, Mitchell L. The Role of Peer Networks in Leadership Development for AHPs. Br J Healthc Leadersh. 2020;26(1):25–31.

[18] O’Leary D, Allen M. Building Leadership Capacity through Fellowship Programmes: Perspectives from Allied Health Professionals. J Health Leadersh Manag. 2022;14(2):112–26.

[19] Kolb DA. Experiential learning: Experience as the source of learning and development. Englewood Cliffs (NJ): Prentice-Hall; 1984.

[20] Wenger E. Communities of practice: Learning, meaning, and identity. Cambridge: Cambridge University Press; 1998.

[21] Pawson R, Tilley N. Realistic evaluation. London: SAGE; 1997.

[22] Wright D et al. Building research capacity in musculoskeletal health: qualitative evaluation of a graduate nurse and allied health professional internship programme. BMC Health Serv Res. 2020;20(1):751. Available from: 10.1186/s12913-020-05628-1 10.1186/s12913-020-05628-1 PMC7429677 32799869

[23] Haynes A, Gilchrist H, Oliveira JS, Tiedemann A. Using realist evaluation to understand process outcomes in a COVID-19-impacted yoga intervention trial: a worked example. Int J Environ Res Public Health. 2021;18(17):9065. 34501654 10.3390/ijerph18179065 PMC8431647

[24] Wiltshire G, Ronkainen N. A realist approach to thematic analysis: making sense of qualitative data through experiential, inferential and dispositional themes. J Crit Realism. 2021;20(2):159–80. 10.1080/14767430.2021.1894909.

[25] Wenke RJ, et al. Allied health research positions: a qualitative evaluation of their impact. Health Res Policy Syst. 2017;15(1):6. 10.1186/s12961-016-0166-4. 28166817 10.1186/s12961-016-0166-4 PMC5292788

[26] Ruslin R, Mashuri S, Rasak MSA, Alhabsyi F, Syam H. Semistructured Interview: A methodological reflection on the development of a qualitative research instrument in educational studies. IOSR J Res Method Educ. 2022;12(1):22–9.

[27] Braun V, Clarke V. Using thematic analysis in psychology. Qual Res Psychol. 2006;3(2):77–101.

[28] Victoria Clarke & Virginia Braun. Thematic analysis. J Posit Psychol. 2017;12(3):297–8. 10.1080/17439760.2016.1262613.

[29] Espedal G, Løvaas BJ, Sirris S, Wæraas A, editors. Researching Values: Methodological Approaches for Understanding Values Work in Organisations and Leadership. Cham: Palgrave Macmillan; 2022. 10.1007/978-3-030-90769-3.

[30] Lincoln Y, Guba EG. Naturalistic inquiry. Newbury Park (CA): Sage; 1985.

[31] O’Connor C, Joffe H. Intercoder Reliability in Qualitative Research: Debates and Practical Guidelines. Int J Qual Methods. 2020;19:1609406919899220. 10.1177/1609406919899220.

[32] Billett S. Learning through practice: Models, traditions, orientations, and approaches. Dordrecht: Springer; 2011.

[33] DeRue DS, Wellman N. Developing leaders via experience: The role of developmental challenge, learning orientation, and feedback availability. J Appl Psychol. 2009;94(4):859–75. 10.1037/a0015317. 19594230 10.1037/a0015317

[34] Angus RL, Hattingh HL, Weir KA. Experiences of hospital allied health professionals in collaborative student research projects: a qualitative study. BMC Health Serv Res. 2022;22(1):729. 10.1186/s12913-022-08119-7. 35650578 10.1186/s12913-022-08119-7 PMC9161454

[35] NHS England. The NHS Long Term Plan. 2019 [cited 2025 May 1]. Available from: https://www.longtermplan.nhs.uk/wp-content/uploads/2019/08/nhs-long-term-plan-version-1.2.pdf

[36] Frenk J, Chen L, Bhutta ZA, Cohen J, Crisp N, Evans T, et al. Health professionals for a new century: Transforming education to strengthen health systems in an interdependent world. Lancet. 2010;376(9756):1923–58. 10.1016/S0140-6736(10)61854-5. 21112623 10.1016/S0140-6736(10)61854-5

[37] Day DV, Fleenor JW, Atwater LE, Sturm RE, McKee RA. Advances in leader and leadership development: A review of 25 years of research and theory. Leadersh Q. 2014;25(1):63–82. 10.1016/j.leaqua.2013.11.004.

[38] NHS Improvement. Developing people – improving care: A national framework for action on improvement and leadership development in NHS-funded services. 2016 [cited 2025 May 1]. Available from: https://eoe.leadershipacademy.nhs.uk/wp-content/uploads/sites/31/2019/04/Developing_People-Improving_Care-010216.pdf

[39] NHS England. National Allied Health Professionals Preceptorship and Foundation Support Programme. 2023 [cited 2025 May 1]. Available from: https://www.hee.nhs.uk/our-work/allied-health-professions/education-employment/national-allied-health-professionals-preceptorship-foundation-support-programme

